# Congenital nephrogenic diabetes insipidus presenting as osmotic demyelination syndrome in infancy: A case report

**DOI:** 10.1097/MD.0000000000028552

**Published:** 2022-01-21

**Authors:** Satoru Kobayashi, Nana Mizuno, Kyoko Yokoi, Takayasu Mori, Eisei Sohara, Shinichi Uchida

**Affiliations:** a Department of Pediatrics, Nagoya City University West Medical Center, Nagoya, Japan; b Department of Nephrology, Graduate School of Medical and Dental Sciences, Tokyo Medical and Dental University, Tokyo, Japan.

**Keywords:** AVPR2, hypernatremia, nephrogenic diabetes insipidus, osmotic demyelination syndrome

## Abstract

**Rationale:**

Almost 90% of congenital nephrogenic diabetes insipidus (NDI) cases are caused by mutations in the arginine vasopressin receptor 2 gene, which has X-linked recessive inheritance. Although NDI is commonly diagnosed in early infancy based on its characteristic findings, clinical diagnosis can be delayed when no other family members have been diagnosed with NDI because several findings of NDI are nonspecific.

**Patient concerns:**

A 3-month-old boy diagnosed with NDI presenting with osmotic demyelination syndrome (ODS) was admitted for poor weight gain after birth and poor feeding during the week prior to admission.

**Diagnosis:**

On admission, the initial blood examination showed hypernatremia (158 mmol/L), and treatment with intravenous fluids over the next 2 days further elevated the serum sodium level (171 mmol/L). After admission, polyuria was recognized, and polyuria in his grandmother and mother since childhood without a diagnosis of NDI was found. Magnetic resonance imaging showed multifocal, symmetrical lesions, including the lateral pons, on diffusion- and T2-weighted imaging, which led to a diagnosis of ODS.

**Intervention:**

The infusion was stopped, and the patient was fed milk diluted 2-fold with water.

**Outcomes:**

The serum sodium level gradually decreased to 148 mmol/L over the course of 1 week. Low-sodium milk was started at 4 months of age and maintained a serum sodium level of approximately 140 mmol/L, which was within the normal range. The developmental quotient was 94 at 4 years of age.

**Lessons:**

ODS is an encephalopathy resulting from extreme fluctuations in serum sodium concentration and plasma osmolality. ODS due to hypernatremia has been reported in several patients, although it usually occurs during rapid correction of hyponatremia. Consequences of the central nervous system are a critical complication of NDI that affects prognosis. These consequences can be avoided with treatment. Early blood examination or polyuria in the patient, mother, or another family member and hypernatremic dehydration with good urine output should lead to an early diagnosis and prevent central nervous system consequences.

## 1 Introduction

Nephrogenic diabetes insipidus (NDI) is a rare congenital and acquired disease. It is characterized by an inability to concentrate urine due to the insensitivity of the collecting tubules to arginine vasopressin.^[1]^ Common causes of congenital NDI are mutations in the arginine vasopressin receptor 2 (AVPR2) gene, which has X-linked recessive inheritance. Other congenital cases result from mutations in the aquaporin 2 gene, which can have an autosomal recessive or dominant inheritance.^[1,2]^ Although NDI is commonly diagnosed in early infancy based on its characteristic findings, such as polyuria, polydipsia, fever of unknown etiology, convulsions, vomiting, and constipation,^[3]^ diagnosis of mild cases of NDI can be delayed. Osmotic demyelination syndrome (ODS), which includes both central pontine myelinolysis and extrapontine myelinolysis, is an encephalopathy that results from extreme fluctuations in serum sodium concentration and plasma osmolality.^[4]^ Although it usually occurs during rapid correction of hyponatremia, ODS due to hypernatremia has been reported in several patients, mainly adults, who develop acute hypernatremia due to various etiologies.^[5–7]^ Only few cases of ODS in infants have been reported.^[8]^ Here, we present the case of an infant with ODS resulting from hypernatremia due to NDI.

## 2 Case report

**Figure 1:**
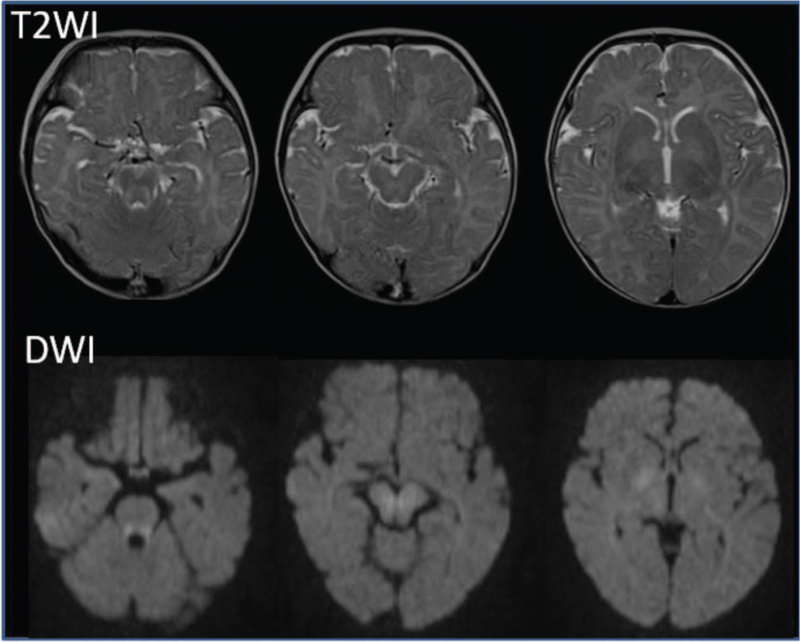
Initial MRI on the third hospital day shows multifocal, symmetric lesions within the superior cerebellar peduncle, lateral pons, thalami, posterior limb of internal capsule on DWI and T2-weighted image. DWI = diffusion-weighted imaging, MRI = magnetic resonance imaging.

A 3-month-old boy was admitted with poor weight gain after birth and poor feeding without vomiting or fever. He was born at full term with a birth weight of 3094 g and no complications during pregnancy. His grandmother and mother had polyuria since childhood without a diagnosis of NDI. The patient's older sister was healthy and had no polyuria. The patient was not examined until admission, although his poor body weight gain had been followed up at a local clinic from approximately 1 month of age. The week before admission, his weight decreased from 4880 to 4770 g and he was feeding poorly. At presentation, the initial blood examination showed hypernatremia (158 mmol/L) and elevated serum chloride (125 mmol/L), uric acid (8.9 mg/dL), and blood urea nitrogen (BUN; 25 mg/dL) levels, which suggested severe dehydration. Table 1 shows the results of blood analysis and infusion. Isotonic fluid containing 90 mEq/L of sodium were given 20 mL/kg/h for the first 2 hours (3.7 mEq/kg/2 h), followed by hypotonic fluid (sodium 35 mEq/L) corresponding to sodium 2.6 mEq/kg/18 h. The second blood examination, performed 20 hours after fluid therapy started, showed decreased BUN (15.1 mg/dL) but elevated natremia (161 mmol/L). Subsequently, fluid therapy was changed to isotonic fluid containing 140 mEq/L of sodium and continued to the next day (sodium corresponding to 21 mEq/kg/24 h). Treatment with intravenous fluids over the next 2 days further elevated the serum sodium (171 mmol/L) while decreasing the uric acid (7.1 mg/dL) and BUN (13.4 mg/dL). After admission, polyuria with a urine volume of 800 to 1000 mL/d (3041-3846 mL/m^2^/d) was observed. Based on the polyuria and high serum sodium levels, diabetes insipidus was suspected. On the third day of admission, a high serum ADH level (130 pg/mL), plasma osmolality of 350 mOsm/kg, and low urine osmolality of 136 mOsm/kg led to a diagnosis of NDI. Magnetic resonance imaging (MRI) showed multifocal, symmetrical lesions in the superior cerebellar peduncle, lateral pons, thalami, and posterior limb of the internal capsule on diffusion-weighted imaging (DWI) and T2-weighted imaging, which led to the diagnosis of ODS (Fig. 1), although no abnormalities were found in the pituitary. No central nervous system (CNS) findings of ODS, such as lethargy, convulsions, and coma, were recognized, except for poor feeding during the week before admission, which might be related to NDI in addition to ODS.

**Table 1:** Blood analysis during initial treatment.

Day from admission	Sodium (mEq/L)	Chloride (mEq/L)	UA (mg/dL)	BUN (mg/dL)	Creatinine (mg/dL)	Serum osmolality (mOsm/kg)	Interventions
On admission	158	125	8.9	25.0	0.46	ND	Isotonic fluid (90 mEq/L of sodium) were given for the first 2 h (3.7 mEq/kg/2 h), followed by hypotonic fluid (sodium 35 mEq/L) corresponding to sodium 2.6 mEq/kg/18 h.
1d (20 h)	161	125	7.2	15.1	0.44	ND	Isotonic fluid (140 mEq/L of sodium) continued to next day (sodium corresponding to 21 mEq/kg/24 h).
2 d	171	137	7.1	13.4	0.48	350	Infusion was stopped, milk diluted 2-fold with water orally or by tube.
4 d	166	131	7.4	15.2	0.57	347	
6 d	156	121	6.4	20.5	0.47	326	
8 d	154	120	ND	17.5	0.41	323	
10 d	148	112	5.1	16.1	0.35	ND	

After the patient was fed milk diluted 2-fold with water orally or by tube, and after the infusion was stopped, the serum sodium level gradually decreased to 148 mmol/L over 1 week. Low-sodium milk was started at 4 months of age and maintained a serum sodium level of approximately 140 mmol/L, which was within the normal range. After the diagnosis, the patient was unable to consume sufficient oral milk and required tube feeding. However, frequent vomiting occurred when a nasogastric tube was used, suggesting gastroesophageal reflux, necessitating a gastroduodenal tube until 16 months of age. Subsequently, he had difficulty eating orally until 2 years of age, necessitating a nasogastric tube. Treatment with trichlormethiazide started at 14 months of age, which decreased urine volume by 30%.

**Figure 2:**
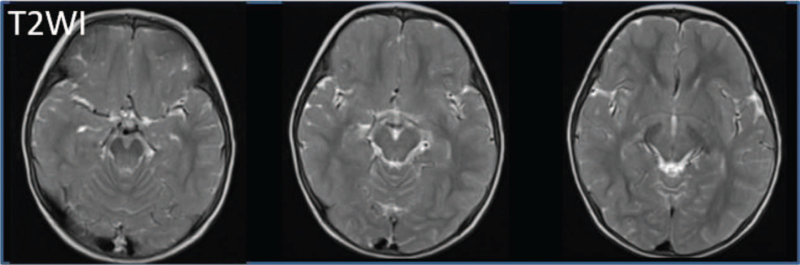
Follow-up MRI performed on 1 yr and 8 mo old shows symmetric high signal intensity changes in the superior cerebellar peduncle, lateral pons, thalami, posterior limb of internal capsule on coronal T2-weighted image. MRI = magnetic resonance imaging.

Follow-up MRI performed at 20 months of age revealed high signal intensity changes in the lateral pons and bilateral thalami on T2-weighted images, which normalized on DWI (Fig. 2). At 4 years of age, he was 94.1 cm (–1.5 SD) tall and weighed 13.8 kg (–0.9 SD). His urine volume was approximately 3 L per day. He had no hydronephrosis and his motor development was almost normal. The assessment of the developmental quotients was 94, performed using the Kyoto Scale of Psychological Development test, as previously described.^[9]^

Genomic DNA was extracted from peripheral blood leukocytes of the patient and his parents. The sequence of the AVPR2 coding region of the proband revealed a reported deletion, c.835_837delGTC [p.Val279del].^[10]^ His mother was heterozygous for this deletion.

## 3 Discussion

Nephrogenic diabetes insipidus is a rare condition. Mutations in AVPR2 cause 90% of congenital NDI cases and occur at a frequency of 4 to 8 per 1 million live male births.^[1]^ NDI is commonly diagnosed based on characteristic findings such as polyuria, polydipsia, fever of unknown etiology, convulsions, vomiting, and constipation in early infancy.^[3]^ The diagnosis of NDI is often delayed and intellectual disability may occur as a consequence of delayed treatment.^[1]^ In our case, the diagnosis of NDI was delayed because of a lack of other signs, such as vomiting and fever, although poor body weight gain was recognized after birth.

Intellectual disability is a major complication of NDI and critically affects the prognosis. The reported frequency of intellectual disability as a consequence of NDI ranges from 70% to 90%,^[11]^ although several papers reported that the majority of patients with NDI have normal intelligence.^[3]^ Intellectual disability can be avoided through treatment. NDI leads to recurrent severe hyperosmotic dehydration and brain edema caused by attempts to rehydrate too quickly.^[12]^ Such complications are mostly caused by de novo mutations, when the clinical diagnosis can be delayed.^[13]^

The term ODS is now preferred to the original, more restrictive terms central pontine myelinolysis and extrapontine myelinolysis.^[14]^ ODS is an encephalopathy resulting from extreme fluctuations in serum sodium concentration and plasma osmolality.^[4]^ It is associated with alcoholism, malnutrition, prolonged diuretic use, psychogenic polydipsia, burns, liver transplantation, postpituitary surgery, and post-urological surgery/gynecological surgery, especially in cases involving glycine infusions.^[14]^ ODS usually occurs during the rapid correction of hyponatremia, whereas it rarely occurs in severe hypernatremia.^[5–8]^ Experimental work in laboratory animals with induced hypernatremia has revealed cellular damage and myelinolysis. Acute hypernatremia is characterized by cellular dehydration due to a shift of water from the interstitial and intracellular compartments. In the presence of sustained hypernatremia, there is an increase in idiogenic osmolytes in the brain, which increases cerebral osmolality and reduces osmotic imbalances.^[6]^

In our case, although MRI performed on the third day showed signs of ODS, these findings were also seen in the T2-weighted images and DWI, which implied that the subacute phase of ODS occurred up to 1 week earlier, when poor feeding might have developed due to fluctuations in the serum sodium concentration and plasma osmolality. Brown suggested that the risk for ODS was greatest when a too rapid or large correction of hypernatremia was performed, and that it must not exceed 0.5 mmol/L/h or more than 10 mmol/L/d in chronic hypernatremia.^[15]^ The changes in the serum sodium levels in our case did not exceed these ranges after admission. However, the possibility that inappropriate intravenous fluid therapy over 2 days after admission leads to CNS involvement cannot be excluded.

The symptoms and signs of ODS, which are often irreversible or only partially reversible, include dysarthria, dysphagia, paraparesis or quadriparesis, behavioral disturbances, movement disorders, seizures, lethargy, confusion, disorientation, obtundation, and coma.^[14]^ Our patient had no remarkable CNS findings at presentation, such as seizures, lethargy, and coma. At 4 years of age, his motor development was almost normal, with no paraparesis, movement disorder, or intellectual disability. Poor oral feeding at the time of ODS diagnosis may be associated with CNS lesions due to ODS. However, we believe that gastroesophageal reflux that leads to frequent vomiting occurs due to NDI rather than ODS because the voluminous amounts of water kept in patients’ stomachs would exacerbate physiologic gastrointestinal reflux in infants and toddlers.^[2]^ There are reports of gastrointestinal disturbance in patients with NDI, 34% of whom require tube feeding or gastrostomy. Tube feeding was discontinued at a median age of 2 years once growth failure resolved, and oral intake was deemed adequate.^[16]^

Several NDI findings are nonspecific. Poor body weight gain can be the only finding in cases of NDI, especially in early infancy, when no other family members have been diagnosed with NDI. Blood examination or polyuria in the mother or another family member should lead to an early diagnosis. Hypernatremic dehydration in a patient with good urine output must raise the consideration for NDI with consequent measurement of urine osmolality and instigation of appropriate fluid treatment, which could avoid inappropriate fluids and prevent CNS consequences.

## Author contributions

**Conceptualization:** Satoru Kobayashi.

**Data curation:** Nana Mizuno

**Investigation:** Takayasu Mori, Eisei Sohara, and Shinichi Uchida.

**Writing – original draft:** Satoru Kobayashi.

**Writing – review & editing:** Nana Mizuno, Kyoko Yokoi.
